# Field survey data for conservation: Evaluating suitable habitat of Chinese pangolin at the county‐level in eastern China (2000–2040)

**DOI:** 10.1002/ece3.11512

**Published:** 2024-06-03

**Authors:** Wei Liu, Xiaoxiao Nie, Fengjiao Chen, Ning Guo, Yong Zhang, Shuping Xiao, Yanbin Huang, Yanping Xie

**Affiliations:** ^1^ College of Life Sciences Henan Normal University Xinxiang Henan China; ^2^ Luoyuan National Forest Farm in Fujian Province Fuzhou Fujian China; ^3^ Wildlife Protection Center of Fujian Province Fuzhou Fujian China; ^4^ Fujian Institute of Forest Inventory and Planning Fuzhou Fujian China; ^5^ Mingxi Forestry Bureau Sanming Fujian China; ^6^ Fujian Junzifeng National Nature Reserve Management Bureau Sanming Fujian China; ^7^ College of Life Sciences Huaibei Normal University Huaibei Anhui China

**Keywords:** Chinese pangolin, conservation, county‐level scale, population dynamics

## Abstract

The scarcity of up‐to‐date data on the distribution and dynamics of the Chinese pangolin (*Manis pentadactyla*) presented a significant challenge in developing effective conservation strategies and implementing protective measures within China. Currently, most of China's national‐level nature reserves and administrative departments operate at the county level, thereby limiting the applicability of larger‐scale analyses and studies for these administrative entities. This study employed 11 widely used modeling techniques created within the Biomod2 framework to predict suitable habitats for the pangolin at the county scale, while examining the correlation between environmental variables and pangolin distribution. The results revealed that highly suitable habitats in Mingxi County of China encompassed only 49 km^2^. Within the county‐managed nature reserve, the proportion of highly suitable habitats reached as high as 52%. However, nearly half of these areas, both moderately and highly suitable habitats, remained inadequately addressed and conserved. We found nine administrative villages that necessitated prioritized conservation efforts. The study anticipated an overall expansion in suitable habitats over the ensuing two decades, with significant growth projected in the eastern regions of Xiayang and Hufang Town. This research offered a clear and applicable research paradigm for the specific administrative level at which China operates, particularly pertinent to county‐level jurisdictions with established nature reserves.

## Graphical abstract

This research offered a clear and applicable research paradigm for the specific administrative level at which China operates, particularly pertinent to county‐level jurisdictions with established protected areas. Given the constraints of the existing data and in order to more precisely evaluate the pangolin's situation at the county scale, the study underscored the paramount importance of conducting field surveys, deemed as the most urgent task at the time.

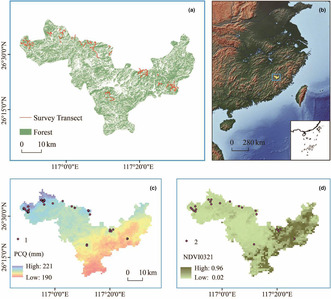

## 1 INTRODUCTION

The Chinese pangolin (*Manis pentadactyla*), an endemic scaly mammal unique to Asia, has attracted significant global focus due to its distinct biological attributes and the grave threats to its existence (Wang et al., 2020; Yan et al., 2021; Zhang et al., 2021). Serving as a myrmecophagous organism, it plays an integral role in the regulation of termite and ant populations (Li et al., 2011). Nevertheless, this species confronts substantial survival challenges, primarily attributed to illicit poaching and habitat degradation (Challender et al., 2020; Heinrich et al., 2016; Wu et al., 2002). Factors such as the illegal trade (Gu, Hu, & Yu, 2023; Nash et al., 2018; Shirley et al., 2023) and local consumption of pangolin meat (Emogor et al., 2023) are posited as the principal motivators behind its poaching. Presently, the population of the Chinese pangolin has diminished by a staggering 90%, leading to its classification as critically endangered (CR) by the International Union for Conservation of Nature (IUCN) (Challender et al., 2019), inclusion in Appendix S1 of the Convention on International Trade in Endangered Species of Wild Fauna and Flora (CITES), and designation as a first‐class protected species under the national conservation laws of China (Notice No. 3, 2021, National Forestry and Grassland Administration, Ministry of Agriculture and Rural Affairs, http://www.forestry.gov.cn/). The prospects for this species are rather bleak (Bashyal et al., 2021; Yang et al., 2018), and the deficiency of contemporary data regarding its population distribution and dynamics poses a pressing challenge in the formulation and execution of conservation strategies and actions (Hu et al., 2010; Kong et al., 2021; Sharma, Rimal, et al., 2020).

Recent studies have elucidated that the Chinese pangolin (*Manis pentadactyla*) predominantly inhabits the southeastern territories of China (Ta et al., 2021). The Wuyi Mountain region is identified as the most pivotal habitat for this species in eastern China (Peng, 2020; Yang et al., 2018; Zhou, 2022). Furthermore, the distribution of the Chinese pangolin is significantly influenced by human activities and variations in precipitation (Ta et al., 2021; Yang et al., 2018), providing crucial support for comprehending its current distributional status. However, the predominance of county‐level units in China's national‐level protected areas presents a limitation in conducting analyses at larger scales, notably at the provincial level and above, thus diminishing their practical utility for administrative departments. Consequently, meticulous analyses at the county level are imperative for formulating viable and effective conservation strategies. A grave challenge encountered globally is the dearth of dedicated protected areas with a primary focus on pangolin conservation (Katuwal et al., 2017; Sharma, Sharma, et al., 2020; Wei et al., 2022). The existing sanctuaries lack targeted scope and specificity in policy development (Nash et al., 2016; Sharma, Rimal, et al., 2020). Therefore, a detailed examination of environmental influences such as climatic conditions, geological factors, and anthropogenic disturbances on the Chinese pangolin at finer scales, coupled with predictions of potential suitable habitats, is essential. Such research will not only deepen our understanding of the local population dynamics and distribution of the Chinese pangolin but also furnish administrative entities with direct and efficacious scientific underpinnings.

Sanming City, located in the eastern segment of the Wuyi Mountain Range in southeastern China, is distinguished for its abundant biodiversity and unique natural habitat, historically constituting a critical distribution zone for the Chinese pangolin (Zhou, 2022). Despite its ecological significance, comprehensive scientific studies pertaining to the population distribution and dynamics of the Chinese pangolin in this area are markedly lacking. This investigation aims to forecast the potential distribution zones of the Chinese pangolin in Mingxi County, Sanming City, leveraging field survey data, Geographic Information Systems, remote sensing technologies, and the Biomod2 model, for both the present and the upcoming two decades. The varied topography and extensive vegetation varieties in Mingxi County provide prospective habitats for the pangolin. The objective of this study is to elucidate the correlation between environmental variables and pangolin distribution and to predict potential suitable habitats. This is intended to supply actionable scientific recommendations for local policymakers and serve as a paradigm for formulating conservation policies for endangered species, like the Chinese pangolin, in national, provincial, and county‐level protected areas throughout China. In light of the global imperative for biodiversity conservation and the practical demands of wildlife protection, this research emphasizes the significance of engaging in detailed, scientific investigations at a granular scale within the field of wildlife conservation.

This research is of paramount importance for county‐level administrative and management entities in China, particularly in the context of developing conservation strategies for endangered species like the Chinese pangolin. Our methodology encompassed a series of crucial steps to fulfill the objectives: Initially, detailed location data for the Chinese pangolin were amassed through extensive field expeditions. Subsequently, environmental datasets were meticulously gathered and rigorously corrected to assure their accuracy. Furthermore, an assessment was conducted on the alterations in suitable habitats, both in the current scenario and projected over the next two decades, including an analysis of their potential influencing elements. Lastly, with a consideration of the demarcations of protected zones and the perimeters of administrative villages, tailored conservation proposals were formulated for immediate and long‐term implementation. These strategic approaches substantially elevate the study's reliability and utility, solidifying its vital role in the formulation of local conservation policies.

## 2 METHODS

### 2.1 Study area

**FIGURE 1:**
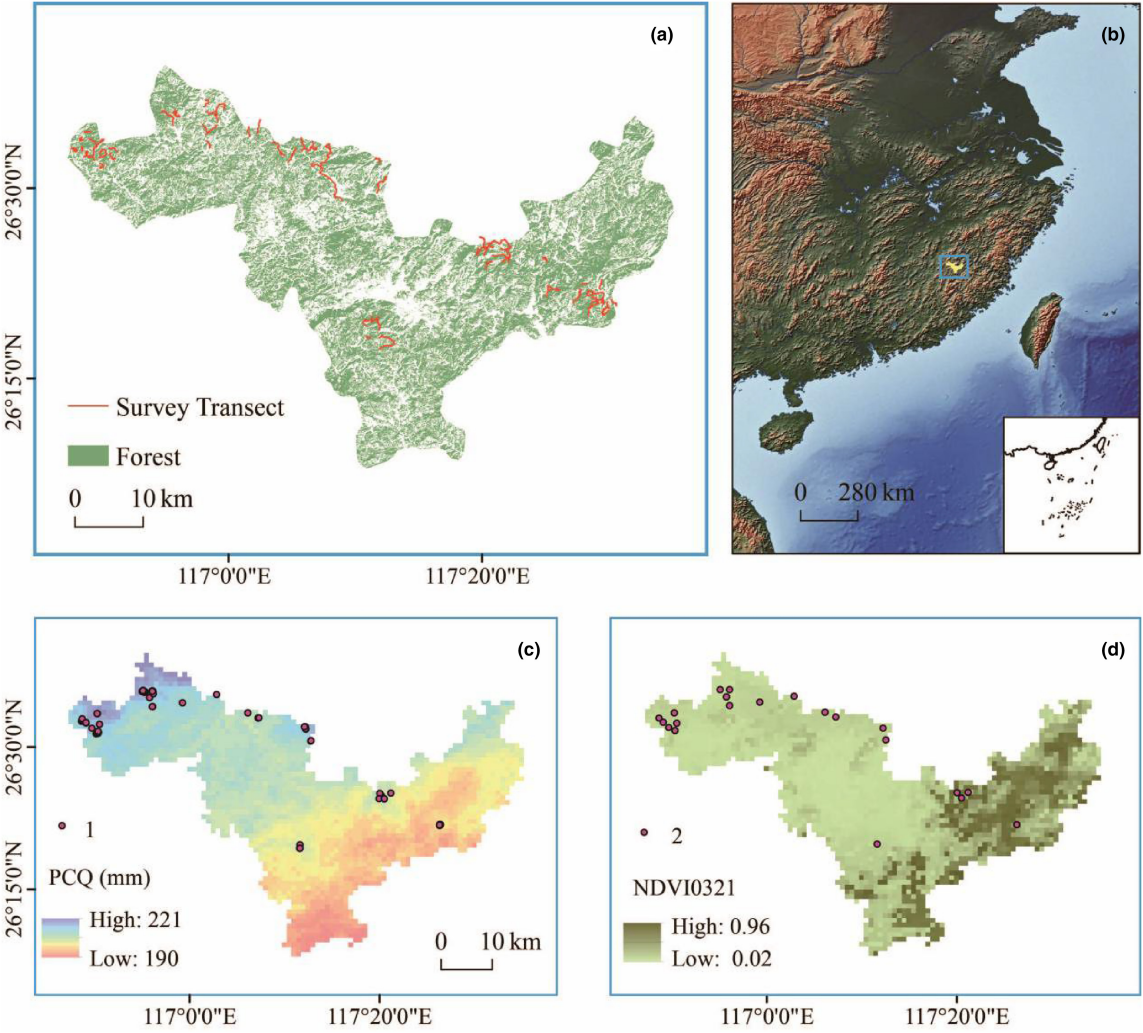
Spatial representation of Mingxi County's research zone and *Manis pentadactyla* (Chinese pangolin) burrow sites. Panel a delineates Mingxi County within Sanming City, Fujian Province, China, highlighting the strategic deployment of 90 ecological transects for this study. Panel b contextualizes the study area within the Southern China geographical zone. In Panel c, marked by numeral “1,” all identified pangolin burrow sites are pinpointed, derived from comprehensive field expeditions (“Burrow site through Investigation”), with “PCQ Value” indicating the quantified precipitation during the coldest climatic quarter, Bio19. Panel d, denoted by numeral “2,” displays the locations subjected to a stringent 500‐m distance filtration criterion, where “NDVI0321” reflects the Normalized Difference Vegetation Index for Mingxi County in March 2020, serving as an indicator of vegetation vigor and phenological state during that period.

The Wuyi Mountain range, notably its eastern extension, Mingxi County, with a total area of 1730 km^2^, is recognized as an ecologically significant potential habitat for the Chinese pangolin. In the 1980s and 1990s, Sanming City, the administrative region of our study area, served as a significant distribution area for the Chinese pangolin (Yang et al., 2018; Zhou, 1996, 2022), with one Chinese pangolin recorded in the Junzifeng National Nature Reserve in 2021 (Zhou, 2022). Accordingly, this investigation designates Mingxi County in Fujian Province (depicted in Figure 1a,b) as the focal study locale. The county, typified by a subtropical monsoonal ecosystem, averages an annual temperature near 18°C with mean precipitation around 2000 mm (Shi, 2021). An impressive over 80% forest coverage (Zhang & Hunag, 2011) contributes to its biodiverse landscape, previously a stronghold for the pangolin population (Zhou, 1996). Mingxi's encompassing nine townships and the Junzifeng National Nature Reserve (26°19′ N to 26°39′ N, 116°47′ E to 117°31′ E), devoted to the preservation of subtropical evergreen broadleaf biomes and the safeguarding of endemic fauna such as the Cabot's Tragopan, delineate its ecological significance. This research utilized vector data delineating the townships and administrative village boundaries, sourced from the county's environmental governance agencies, to frame the geographical scope of the habitat suitability analysis.

### 2.2 Species distribution data

Due to the nocturnal nature of Chinese pangolins and their low population density, direct studies on individual specimens pose significant challenges (Macdonald, 2006). Therefore, this research adopts an indirect approach, focusing on the analysis of pangolin burrow systems. This methodology is instrumental in elucidating the species' habitat preferences and assessing the environmental determinants influencing their burrow distribution (Sharma, Sharma, et al., 2020; Thapa et al., 2014; Wu et al., 2002), thereby providing critical insights for targeted conservation interventions. In Mingxi County, a methodical stratified random sampling framework was applied, deploying 90 transects across diverse ecological niches: 21 in broadleaf forests, 16 in mixed coniferous‐broadleaf forests, 16 in coniferous forests, 17 in bamboo‐dominated areas, and 20 within agricultural landscapes, each transect extending 1–2 km in length and encompassing a 5–10 m width. Field expeditions were conducted in distinct seasonal windows: November to December 2022, February to March 2023, May to July 2023, and September to October 2023. Geographic coordinates were meticulously documented upon burrow discovery. Given the restricted spatial range of the species, typically confined to less than 1 square kilometer (Sharma, Rimal, et al., 2020), burrows spaced beyond 500 meters were selected for in‐depth analysis. The application of infrared camera traps validated the continued occupancy of these burrows by pangolins. Appendix S1 presents photographic evidence of fresh pangolin burrows (Figure S1), while Appendix S3 presents video and photographs of the Chinese pangolin.

### 2.3 Environmental data

In this study, an integrative modeling approach was applied to assess a spectrum of environmental determinants, stratified into three primary categories: (i) a suite of 19 bioclimatic variables encompassing an array of temperature and precipitation metrics for the period 1970–2000 (https://www.worldclim.org/data/index.html); (ii) topographical and anthropogenic factors, including slope, aspect, altitude, distances from water sources and roads; (iii) temporal dynamics of vegetation health, quantified through the analysis of Normalized Difference Vegetation Index (NDVI) across 23 temporal intervals in 2020. The compilation of these environmental parameters (refer to Table S1 in Appendix S1) provided a robust foundation for habitat suitability modeling.

The environmental variables utilized in this ecological analysis were processed with a 2.5 minutes spatial resolution, standardized to the UTM‐WGS1984 coordinate system. The selection criteria for environmental factors involved a two‐tiered process: preliminary individual factor analysis using the Maxent model, identifying significant contributors (area under curve (AUC) >0.9, contribution rate >10%), followed by a comprehensive collinearity assessment, where one variable from any highly correlated pair (|correlation| >0.8) was omitted (illustrated in Figure S2 in Appendix S1). This procedure culminated in the identification of nine pivotal environmental factors: Bio03, Bio19, NDVI0321, NDVI0727, NDVI0913, Aspect, Roads, Slope, and Waterway, each thoroughly defined in Table S2 in Appendix S1.

### 2.4 Species distribution modeling

1. Biomod2 is a software package based on the R programming platform. It allows for the rapid computation of 10 different species distribution models on a presence/absence dataset and integrate them into ensemble models. Additionally, it includes a model evaluation module that assesses model performance based on metrics like sensitivity, specificity, AUC of receiver operating characteristics (Hanley & McNeil, 1982), and true skill statistics (TSS) (Allouche et al., 2006; Hao et al., 2019). Research on the Giant Panda indicates that Biomod2 has higher predictive accuracy compared to MaxEnt, especially in situations where species distribution points are sparse (Luo et al., 2017). Within the ambit of this study, the Biomod2 software package (Thuiller et al., 2023) was harnessed, amalgamating a cadre of 11 advanced modeling algorithms for a synergistic prediction of species distribution. Utilizing the algorithms integral to Biomod2, a training subset comprising 75% of extant species distribution data was deployed for model calibration, reserving the remaining 25% for model validation purposes. Each algorithm was iterated thrice to fortify the robustness of the results. The pseudo‐absence approach was employed to compensate for the paucity of explicit absence data. Model efficacy was appraised using TSS and the area under the receiver operating characteristic curve (AUC) as metrics, evaluating the precision of model fit. TSS amalgamates sensitivity and specificity, with a score range from −1 to 1, where values between 0.8 to 1 signify optimal model fidelity (Allouche et al., 2006). AUC values span from 0.5 to 1, with thresholds above 0.7 denoting reasonable predictive accuracy, above 0.8 indicating satisfactory predictions, and values surpassing 0.9 reflecting high precision (Anderson, 2003). Models with TSS exceeding 0.7 were integrated to construct the ensemble model, leveraging the EMwmean method, and AUC values were employed as the definitive standard for prediction appraisal.
2. A randomized sampling protocol was applied to ascertain Pearson correlations among all predictive and evaluative variables (Guisan et al., 2017; Thuiller et al., 2023), determining the relative import of each variable in species distribution modeling. This non‐model‐dependent approach allows for streamlined comparisons across different modeling frameworks (Zanardo et al., 2017). Response curves were employed to delineate the gradational changes in species occurrence probability with pivotal predictive variables, elucidating the interplay between species occurrence and environmental drivers, with ecological factors deemed conducive for species survival when the occurrence probability exceeds 0.5.
3. In this research, ArcGIS software was utilized for visual representation of habitat suitability spatial distribution in TIFF formats. Ensemble model outputs dictated the stratification of habitat suitability into four discrete categories: 0–0.15 as unsuitable, 0.15–0.50 as lowly suitable, 0.50–0.75 as moderately suitable, and 0.75–1.00 as highly suitable. Further, an analysis incorporating the perimeter of Fujian Junzifeng National Nature Reserve and current administrative village delineations was conducted. This analysis was pivotal in identifying key administrative villages on the periphery of the reserve, earmarking them as primary zones for conservation and monitoring initiatives. Distribution extents of diverse suitability levels within all administrative villages were methodically ranked, employing a weighted schema (score = highly suitable area × 0.7 + moderately suitable area × 0.5). A conservation benchmark was set to ensure no less than 75% of suitable habitats outside the reserve are conserved, based on which administrative villages necessitating immediate conservation actions were identified. We conducted field surveys in the selected administrative villages, establishing at least one transect in each village to verify the presence of pangolin burrows along the survey lines.
4. For future projections, the BIOMOD_EnsembleForecasting function within Biomod2 was deployed. Predictive variable binary transformation was conducted using ArcGIS's reclassification tool, setting a critical threshold at 0.5, denoting values ≥0.5 as indicative of species presence and < 0.5 as absence. Subsequent comparative analyses of current and projected distributions under the SSP1‐2.6 scenario (similarly for SSP5‐8.5) were facilitated using ArcGIS 10.2. Raster layers were initially reclassified based on habitat suitability, attributing new pixel values. Multiplicative raster calculations were then employed, each pixel value acquiring a novel interpretation: “3” indicating absence, “4” for expansion, “6” for contraction, and “8” for stable regions (He et al., 2018; York et al., 2011). The final phase involved ranking future suitable areas across townships to spotlight regions meriting heightened conservation focus over the next two decades.

All analytical processes were conducted in R software (version 4.3.1, 2023), with spatial analysis executed using ArcGIS (version 10.2; ESRI, Inc., Redlands, CA, USA). Documentation and presentation tasks were facilitated through WPS Office (Kingsoft Office Software, https://www.wps.com/office‐free). The integrated application of these analytical tools endowed the study with robust data processing and analytical prowess, ensuring the accuracy and reliability of the results.

## 3 RESULTS

### 3.1 Model performance analysis

In this ecological study, a comprehensive dataset of 106 Chinese pangolins burrows was collated, with emphasis on analyzing 23 selected burrows in detail (illustrated in Figure 1c,d). Within the scope of the Biomod2 framework, seven predictive models were meticulously chosen, each surpassing the TSS benchmark of 0.7 (as outlined in Table 1). Notably, the Random Forest (RF) and XGBOOST models demonstrated superior Receiver Operating Characteristic (ROC) values of 1, eclipsing the ensemble model's predictive accuracy. Conversely, the Gradient Boosting Machine (GBM), Maximum Entropy (MAXENT), Generalized Linear Model (GLM), Classification Tree Analysis (CTA), and Generalized Additive Model (GAM) yielded ROC values slightly lower than that of the ensemble model (detailed in Table 1). This delineation of results highlights the ensemble model's exceptional proficiency in accurately modeling the distribution patterns of the Chinese pangolin in the near current historical window (1990–2000).

**TABLE 1:** The area under the receiver operating characteristic curve (AUC) and true skill statistics (TSS) values of 10 modeling algorithms used in this study.

Key	Model name	TSS value	AUC value
1	RF	1.00	1.00
2	XGBOOST	1.00	1.00
3	GBM	0.89	0.97
4	MAXENT	0.82	0.90
5	GLM	0.75	0.92
6	CTA	0.71	0.88
7	GAM	0.70	0.86
8	MARS	0.66	0.88
9	FDA	0.57	0.79
10	ANN	0.44	0.73
11	SRE	0.20	0.60
12	EMwmean	0.97	0.99

### 3.2 Assessment of environmental variables in the ensemble model and their impact on species distribution

**FIGURE 2:**
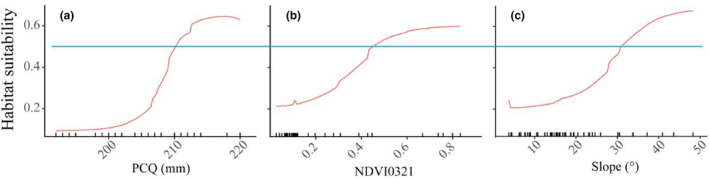
Response Curves for *Manis pentadactyla*'s Predicted Habitat Suitability from the Ensemble Model. Panel a delineates the ecological correlation between the probability of habitat suitability and PCQ (mm) (bio19, quantified precipitation in the coldest quarter), Panel b elucidates the interplay between habitat suitability probability and NDVI0321 (Normalized Difference Vegetation Index for the month of March), and Panel c explicates the relationship between habitat suitability probability and the geomorphological variable of slope. The demarcated blue line represents the critical threshold where the probability equals 0.5, indicating that values exceeding this threshold suggest the ecological factors are optimally conducive to the survival of the species.

In the construct of this study, nine environmental variables were integrated into the predictive modeling framework. Of these, the precipitation during the coldest quarter (bio19), the Normalized Difference Vegetation Index of March (NDVI0321), and the topographic variable of slope emerged as pivotal, each contributing in excess of 10% to the model's predictive capacity, as elucidated in Table 2. This denotes their substantial relevance and influence within the ecological modeling construct. In contrast, the other variables demonstrated a relative importance below the 10% benchmark, suggesting a more marginal role in the model's overall predictive accuracy. Notably, the leading trio of environmental parameters exhibited a significant positive correlation with the probability of habitat suitability for the Chinese pangolin, as illustrated in Figure 2. In comparison, the secondary environmental variables did not exhibit marked correlations with the species' distribution probabilities, underscoring the differential impact of various ecological factors on pangolin habitat suitability.

**TABLE 2:** Importance ranking of factors influencing the distribution of Chinese pangolin, each factor thoroughly defined in Table S2 in Appendix S1.

Environmental factor	Importance (%)	Sorting
Bio19 (PCQ)	58.03	1
NDVI0321	16.5	2
Slope	15.07	3
Waterway	2.86	4
NDVI0913	2.4	5
Bio3	0.63	6
Aspect	0.62	7
NDVI0727	0.43	8
Roads	0.22	9

### 3.3 Habitat suitability distribution analysis in Mingxi County

**FIGURE 3:**
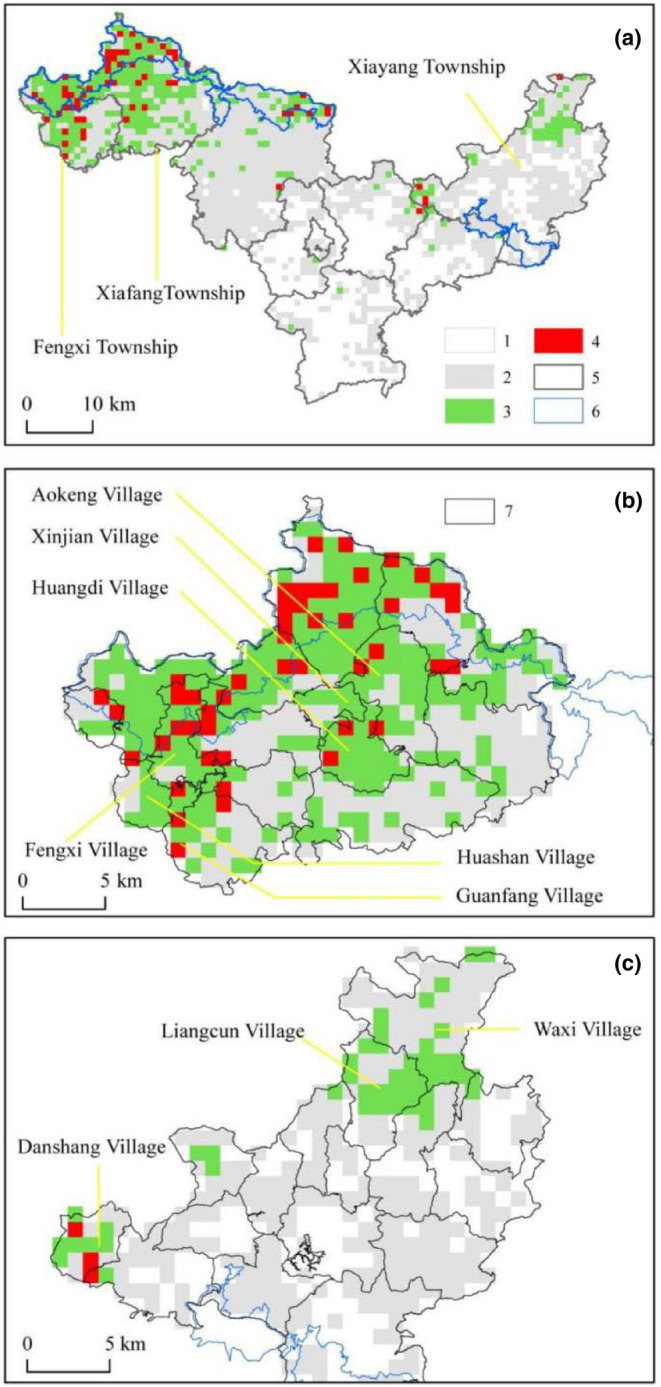
Predictive distribution of *Manis pentadactyla* in Mingxi County, as modeled by the ensemble approach for the contemporary period. Panel a delineates the topographical overview of Mingxi County, identifying various habitat suitability levels: “1” for Unsuitable region, “2” for lowly suitable region, “3” for moderately suitableregion, “4” for highly suitable region, “5” representing the demarcation of township boundaries, and “6” outlining the protected area boundaries. Panel b provides a focused predictive mapping for the western townships, encompassing Fengxi and Xiafang, with “7” indicating the administrative village perimeters and naming three pivotal villages in each township. Panel c visualizes the predictive habitat distribution within Xiayang Township, eastern Mingxi County, highlighting three key administrative villages.

The ensemble model delineated the habitat suitability for the Chinese pangolin in Mingxi County into distinct categories: unsuitable region, encompassing 531 km^2^ (31%) of the total study area; lowly suitable region extending across 890 km^2^ (51%); moderately suitable region covering 260 km^2^ (15%); and highly suitable region, the most critical yet scarce, comprising 49 km^2^, accounting for 3% of the total area. Notably, within the confines of the nature reserve, habitats of highly suitable regions constituted a significant 52% (as depicted in Table 3; Table S3). The spatial analysis revealed a concentration of moderate to high suitability habitats predominantly in the northwestern regions, including Fengxi Township, Xiafang Township, and the eastern sector of Xiayang Township (Figure 3a). A comprehensive ranking of the county's 89 administrative villages was also conducted (Figure 3b), with a detailed exposition of the top nine villages provided in Table 3. We conducted field surveys in the nine administrative villages, all of which exhibited distributions of pangolin burrows (results presented in Figure S3).

**TABLE 3:** Habitat suitability distribution for *Manis pentadactyla* during the near current period within the Fujian Junzifeng National Nature Reserve and in prominent administrative villages surrounding the reserve.

Area name	Unsuitable region (%)	Lowly suitable region (%)	Moderately suitable region (%)	Highly suitable region (%)
Reserve	2	8	28	52
Xiayang Township				
Danshang Village	0	1	2	3
Waxi Village	0	3	4	0
Liangcun Village	0	1	5	0
Fengxin Township				
Guanfang Village	0.00	0.01	0.04	0.05
Huashan Village	0.00	0.00	0.03	0.02
Fengxi Village	0.00	0.00	0.02	0.06
Xiafang Township				
Aokeng Village	0.00	0.01	0.07	0.02
Xinjian Village	0.00	0.00	0.02	0.00
Huangdi Village	0.00	0.00	0.04	0.05
Subtotal	0.00	0.08	0.34	0.22
Total	0.02	0.16	0.62	0.75

*Note*: The table quantifies the distribution percentages of various habitat categories.

### 3.4 Climatic impact on pangolin distribution dynamics

**FIGURE 4:**
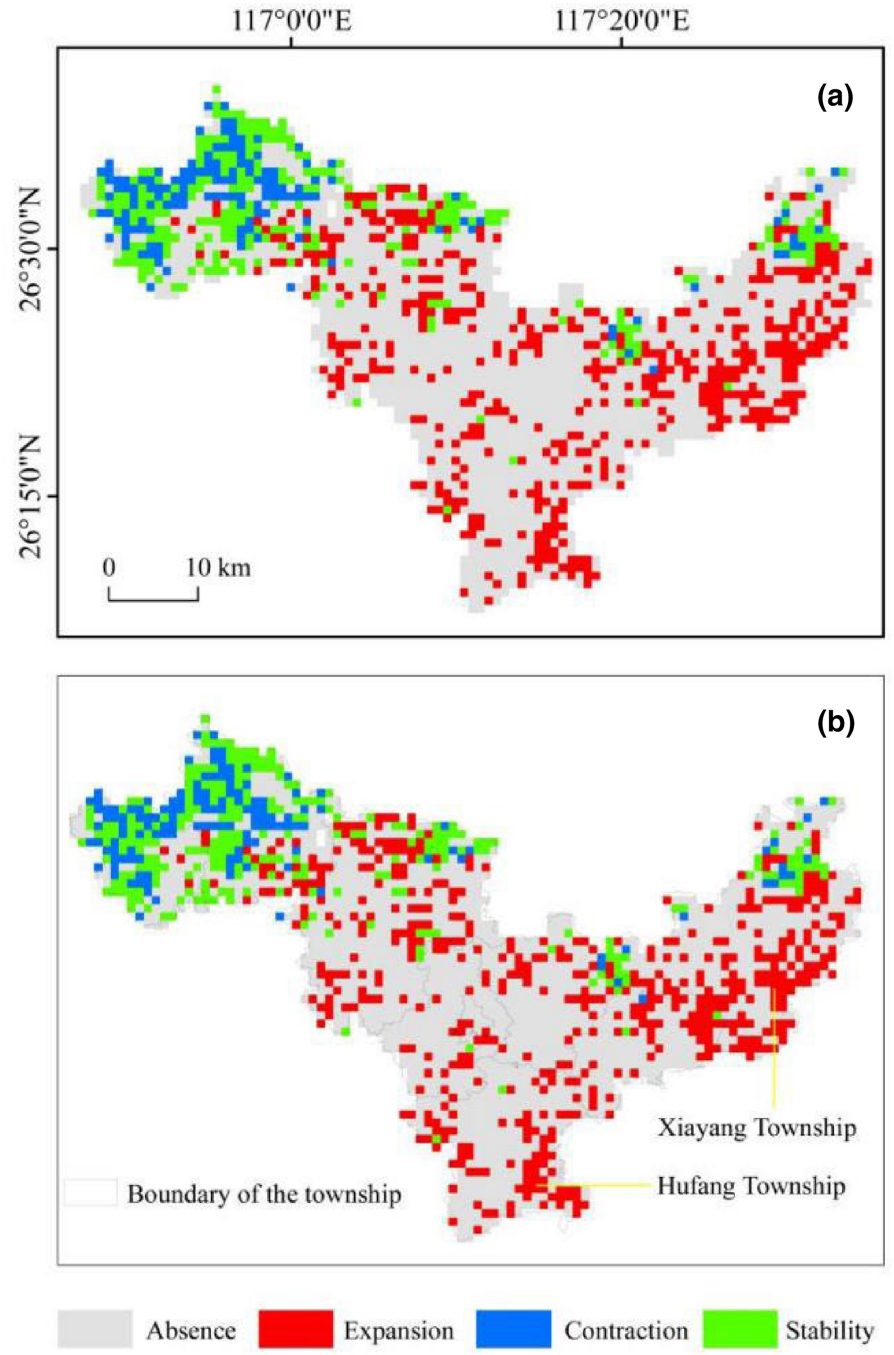
Projected habitat suitability for *Manis pentadactyla* under future climatic scenarios. Panel a represents the habitat distribution under the SSP126 climate model, while Panel b depicts the scenario under SSP585, showing substantial consistency in pangolin habitat distribution between the two models. The habitat contraction is predominantly expected in the western sectors, whereas significant habitat expansion is anticipated in the eastern regions, specifically in Xiayang Township and Hufang Town.

Projections derived from the ensemble model under the SSP1‐2.6 scenario suggest a sustained and increasing presence of the Chinese pangolin habitats in the northwestern areas of Mingxi County through the period 2021 to 2040 (Figure 4a). The SSP5‐8.5 scenario forecasts corroborate these trends (details presented in Figure 4). Under the SSP126 framework, the suitable habitat for the pangolin is projected to undergo an expansion of 378 km^2^, while simultaneously experiencing a contraction of 110 km^2^ by 2040. The contraction is primarily forecasted in western locales, whereas eastern regions, particularly the eastern parts of Xiayang Township and Hufang Town, are anticipated to become focal zones of habitat expansion (Figure 4b).

## 4 DISCUSSION

This research represented a pioneering assessment of the distribution and conservation status of the Chinese pangolin at a detailed county‐level scale, integrating field surveys, remote sensing data, and ecological modeling. This novel approach established a research paradigm for conducting targeted studies on endangered species such as the Chinese pangolin at the county level and devising specific conservation strategies. Our findings indicated that while current protected areas covered 50% of suitable habitats for the pangolin, a significant portion of highly and moderately suitable habitats remained under‐protected. This underscored the urgency of implementing immediate conservation actions in prioritized administrative villages.

### 4.1 Critical environmental variables and rationale

Precipitation emerged as a key environmental driver of the Chinese pangolin distribution in our study. Larger‐scale studies in regions like China and East China have also highlighted a significant positive correlation between precipitation and potential suitable habitats for pangolins (Ta et al., 2021; Yang et al., 2018), suggesting a widespread climatic influence. This link was likely due to the interconnectedness of precipitation with the distribution of pangolin's primary food sources—ants, as well as the association between precipitation patterns and local human activity habits. While other areas might prioritize variables like the mean temperature of the warmest quarter (bio10) and precipitation of the warmest quarter (bio18) (Sharma, Rimal, et al., 2020), our findings highlighted the distinct ecological dynamics at the county scale. Overall, climatic factors played a dominant role in influencing pangolin distribution.

The Normalized Difference Vegetation Index for March (NDVI0321) ranked second in influence, with a contribution rate of 16.5% (see Table 2). Pangolins, primarily feeding on ants and termites, thrived in healthy, dense vegetation (Sharma, Sharma, et al., 2020; Tamang et al., 2022), correlating with higher NDVI values. This suggested a robust dependency on vibrant ecosystems for food sourcing and shelter (Acharya et al., 2021; Sharma, Rimal, et al., 2020; Thapa et al., 2014). Moreover, NDVI indicators reflected the critical ecological period of March, potentially aligning with key pangolin activities such as foraging and breeding. This highlighted the crucial role of maintaining healthy vegetation for the survival of pangolins and other wildlife.

Slope, contributing 15%, was identified as the third most influential factor. This correlation might have been attributed to the improved drainage on sloped terrains, essential for maintaining dry burrows during rainy seasons, and the provision of natural cover against predators. Furthermore, sloped areas typically experience less human disturbance, offering a more stable habitat (Acharya et al., 2021). Our study demonstrated the nuanced relationship between pangolin habitat preferences and topography.

The distances from water sources and roads contribute rates of 2.86% and 0.22%, respectively, which may be attributed to two reasons. First, it could be attributed to the sources of environmental variables. In order to standardize these variables and minimize the potential impact of unstable data on the model, we utilized widely accepted anthropogenic disturbance variables, such as those provided by sources from OpenStreetMap (https://www.openstreetmap.org/), to extract Roads and Waterway. The original data included relatively large county‐level roads and main tributaries of rivers. However, these variables are sparsely distributed locally, diluting their inherent influence on the distribution of the Chinese pangolins. As a result, these major roads and main tributaries contribute less to the potential suitable habitat distribution of the Chinese pangolins in the area. The second reason could be that, compared to the Roads and Waterway, Bio19 (Precipitation of Coldest Quarter) had a greater impact on the potential distribution of the Chinese pangolins.

Although the contribution rates of the distances from water sources and roads are low, they cannot be overlooked. Rivers, as consistent water sources, were vital for pangolin survival. Moist soils near rivers fostered abundant food resources, which are crucial for pangolin sustenance (Katuwal et al., 2017). This factor underscored the importance of riverine habitats in pangolin conservation strategies. Additionally, in the context of illegal poaching, roads facilitate human access, posing a significant threat to the Chinese pangolins, thus making this variable gain importance. Our findings resonated with studies from regions like Dhankuta, Ilam, and Terai in Nepal (Katuwal et al., 2017; Shrestha et al., 2021), where road proximity significantly influenced pangolin distribution. This suggested that roads might serve as critical ecological corridors, highlighting the need for targeted conservation measures.

In summary, this study offered groundbreaking insights into the habitat suitability and conservation needs of the Chinese pangolin, emphasizing the role of climatic, vegetative, topographical, and anthropogenic factors in shaping its habitat preferences. The comprehensive approach taken here sets a precedent for future wildlife conservation research and policy development at a local scale.

### 4.2 Conservation strategy recommendations

In this pioneering study, we utilized an integrative approach combining field surveys, remote sensing data, and ecological modeling, to assess the distribution and conservation status of the Chinese pangolin at a detailed county level. This approach established a groundbreaking framework for research and policy development on endangered species at the county level. Our analysis detailed the proportions of suitable habitat distributions within and outside the nature reserve (refer to Table 3). Notably, nine vital administrative villages outside the reserve accounted for significant portions of moderately and highly suitable habitats. By incorporating these areas into conservation management plans, we were able to effectively cover 62% of moderately suitable and 75% of highly suitable habitats (as illustrated in Figure 3b,c). We recommended the inclusion of these villages in the design of new pangolin reserves and emphasized the importance of policy advocacy and intensified monitoring. Special attention was needed to prevent deforestation and illegal poaching in primary habitats, crucial for the survival of pangolins (Tinsman et al., 2023). Given the significant correlation identified between pangolin consumption and poaching (Bashyal et al., 2021; Emogor et al., 2023; Nash et al., 2016), promoting community awareness and conservation policies in these areas was essential.

Our projections for Mingxi County (2021–2040) indicated an expected increase in the Chinese pangolin's habitat distribution (see Figure 4). This anticipated expansion, likely due to increased precipitation from rising temperatures and positively correlated with pangolin distribution (Ta et al., 2021; Yang et al., 2018). Under China's stringent conservation policies, this habitat expansion offered promise for an increase in the pangolin population. The forecasted habitat extension, particularly in the eastern regions of Mingxi County like Xiayang Township and Hufang Town, underscored the necessity for strategic conservation planning. However, risks posed by global warming and population growth necessitated a careful consideration of these threats (Gao et al., 2022), emphasizing the need for focused monitoring and policy intervention over the next two decades.

This study provided a clear, applicable research model for county‐level administrative units in China, particularly valuable in areas with existing reserves. With the Chinese pangolin being a critically endangered and flagship species, our study's approach aimed to inspire similar conservation efforts across various administrative levels. We encouraged local governments and reserve managers to apply our methodology, significantly contributing to the protection of pangolins and other endangered species. In light of recent discoveries in pangolin species (Gu, Wu, et al., 2023), the urgency of field surveys was emphasized, advocating them as a priority in conservation efforts.

In the future, we recommend gradually conducting relevant work in the surrounding protected areas to identify areas requiring priority protection within different regions. Furthermore, there is currently limited research on the actual population size and ecological functions of pangolins, suggesting the need for further in‐depth studies on population conservation and ecological functionality in the future.

## 5 CONCLUSIONS

This study utilized the Biomod2 integrated model to project potential habitat distributions for the Chinese pangolin at a county‐level scale. Precipitation was identified as the pivotal environmental determinant affecting the distribution of the Chinese pangolin. Within the county‐managed National Nature Reserve, the proportion of highly suitable habitats reached as high as 52%. However, nearly half of these areas, both moderately and highly suitable habitats, remained inadequately addressed and conserved, particularly in nine crucial administrative villages. Habitat expansions for the Chinese pangolin were anticipated, particularly in the eastern areas of Mingxi County. This research not only offered invaluable scientific evidence for the effective preservation of the Chinese pangolin but also served as a clear and applicable research paradigm for the protection of endangered species in regions worldwide at county‐level scale.

## AUTHOR CONTRIBUTIONS

**Wei Liu:** Conceptualization (lead); data curation (lead); formal analysis (lead); funding acquisition (lead); investigation (equal); methodology (lead); project administration (lead); resources (lead); software (lead); supervision (lead); validation (equal); visualization (equal); writing – original draft (lead); writing – review and editing (lead). **Xiaoxiao Nie:** Conceptualization (supporting); data curation (lead); formal analysis (supporting); investigation (equal); methodology (supporting); resources (equal); software (equal); supervision (equal); validation (equal); visualization (equal); writing – original draft (lead); writing – review and editing (lead). **Fengjiao Chen:** Investigation (equal); resources (equal); validation (equal). **Ning Guo:** Investigation (equal); resources (equal); validation (equal); visualization (equal). **Yong Zhang:** Formal analysis (equal); investigation (equal); validation (equal); visualization (equal). **Shuping Xiao:** Investigation (equal); resources (equal); validation (equal); visualization (equal). **Yanbin Huang:** Investigation (equal); validation (equal); visualization (equal). **Yanping Xie:** Conceptualization (lead); data curation (lead); formal analysis (lead); investigation (lead); methodology (lead); project administration (supporting); resources (lead); supervision (lead); validation (lead); visualization (lead); writing – original draft (lead); writing – review and editing (lead).

## CONFLICT OF INTEREST STATEMENT

The authors declare that they have no conflict of interest.

## Supporting information


Appendix S1.

Appendix S2.

Appendix S3.

## Data Availability

Additional supporting information (Appendix S1) may be found on line in the Supporting Information section at the end of the article.
