# Supplementary material for: Field survey data for conservation: Evaluating suitable habitat of Chinese pangolin at the county‐level in eastern China (2000–2040)
Source: Ecol Evol. 2024 Jun 3;14(6):e11512. doi: 10.1002/ece3.11512 (PMC11147814; doi:10.1002/ece3.11512)
Supplement: Supplementary file 1 — Appendix S1. [file ECE3-14-e11512-s002.docx]

Table S1. The environmental factors involved in this study and their data sources.

| category | Varialbe Name | Source |
| --- | --- | --- |
| Climate factor | a suite of 19 bioclimatic variables | WorldClim library (https://worldclim.org/data/index.  html) |
| Topographical factor | slope, aspect and altitude | ASTER GDEM with 30-meter resolution digital elevation data(https://www.gscloud.cn) |
| Anthropogenic factor | hydrological proximity, and infrastructural distance | https://www.openstreetmap.org |
| Vegetation variable | normalized difference vegetation index, NDVI | Resource and Environment Science and Data Center  (https://www.resdc.cn) |

Table S2: Environmental variables and Their Specific Definitions.

| Varialbe Name | Meaning |
| --- | --- |
| Bio03 | Isothermality (BIO2/BIO7) (×100) |
| Bio19 | Precipitation of Coldest Quarter |
| NDVI0321 | NDVI value for March 21, 2020. |
| NDVI0727 | NDVI value for July 27, 2020 |
| NDVI0913 | NDVI value for September 13, 2020 |
| Aspect | aspect is defined as the compass direction that a slope faces. |
| Roads | Distance from roads |
| Slope | Slope gradient is defined as the steepness or incline of a slope, typically measured in degrees from horizontal. |
| Waterway | Distance from waterway |

Table S3: The potential suitable habitat distribution information of Chinese pangolins within the jurisdiction of Mingxi County.

| Town/Area (km^2^) | Unsuitable region | Lowly suitable region | Moderately suitable region | Highly suitable region |
| --- | --- | --- | --- | --- |
| Gaiyang | 12.36 | 299.66 | 35.53 | 6.18 |
| Hufang | 162.96 | 60.24 | 1.54 | 0 |
| Hanxian | 88.04 | 61.79 | 3.86 | 0.77 |
| Chengguan | 90.36 | 50.2 | 0.77 | 0.00 |
| Shaxi | 76.46 | 61.01 | 3.86 | 0.00 |
| Xiayang | 100.4 | 215.48 | 35.53 | 3.09 |
| Fengxi | 0 | 51.75 | 62.56 | 14.67 |
| Xiafang | 0 | 84.96 | 115.08 | 23.94 |
| Xuefeng | 6.18 | 7.72 | 0 | 0 |


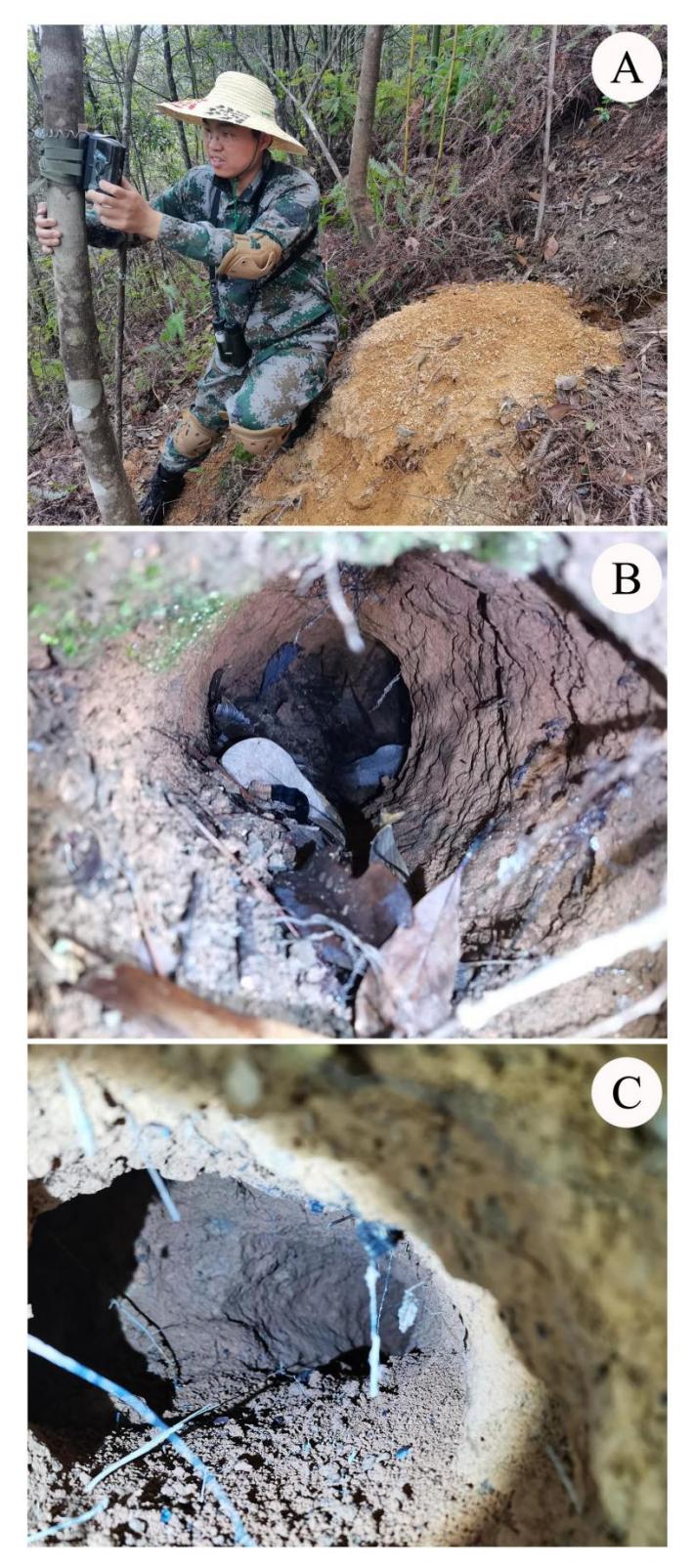


Figure S1. Morphological Features of Chinese Pangolin Burrows. A) Depicts the external appearance of a pangolin burrow characterized by a pronounced earthen mound, alongside the lead author analyzing infrared camera data. B) Illustrates the distinct surface texture on one side of the burrow, exhibiting a characteristic fish-scale pattern. C) Displays the similar fish-scale pattern on the surface of another burrow, highlighting the unique structural attributes of Chinese pangolin burrows.


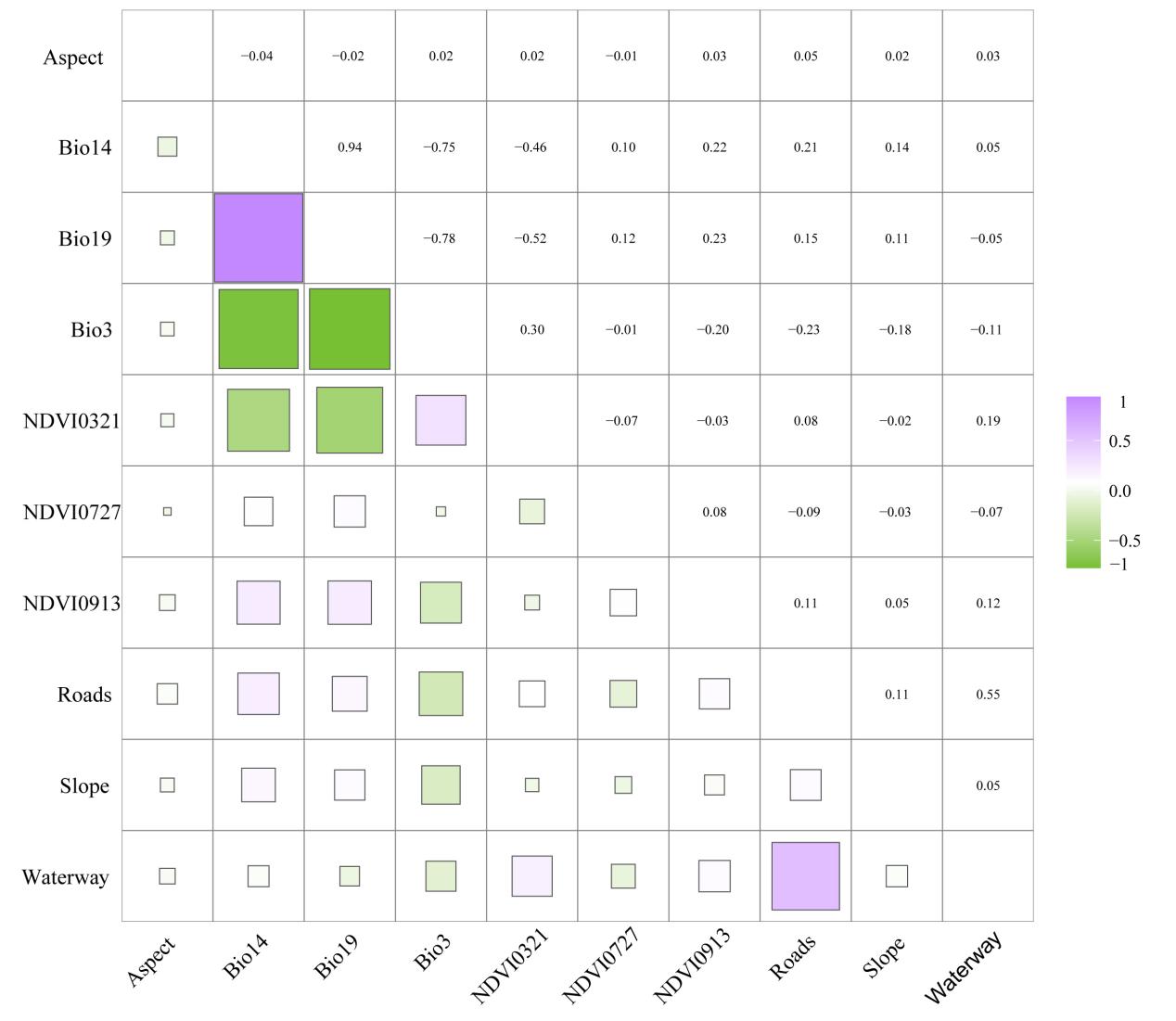


Figure S2. Environmental Variable Correlation Analysis for Chinese Pangolin Burrows in Mingxi County. The upper right section presents the correlation coefficient 'r', ranging from -1 to 1. Positive values indicate a positive correlation, negative values indicate a negative correlation, and a value of 0 indicates no correlation. The absolute value of 'r' reflects the strength of the correlation. The lower left section graphically represents these correlation values. Positive correlations are depicted in purple, and negative correlations in green. The intensity of the color and the size of the circles are proportionate to the magnitude of the correlation coefficients, as elucidated in the right-hand legend.


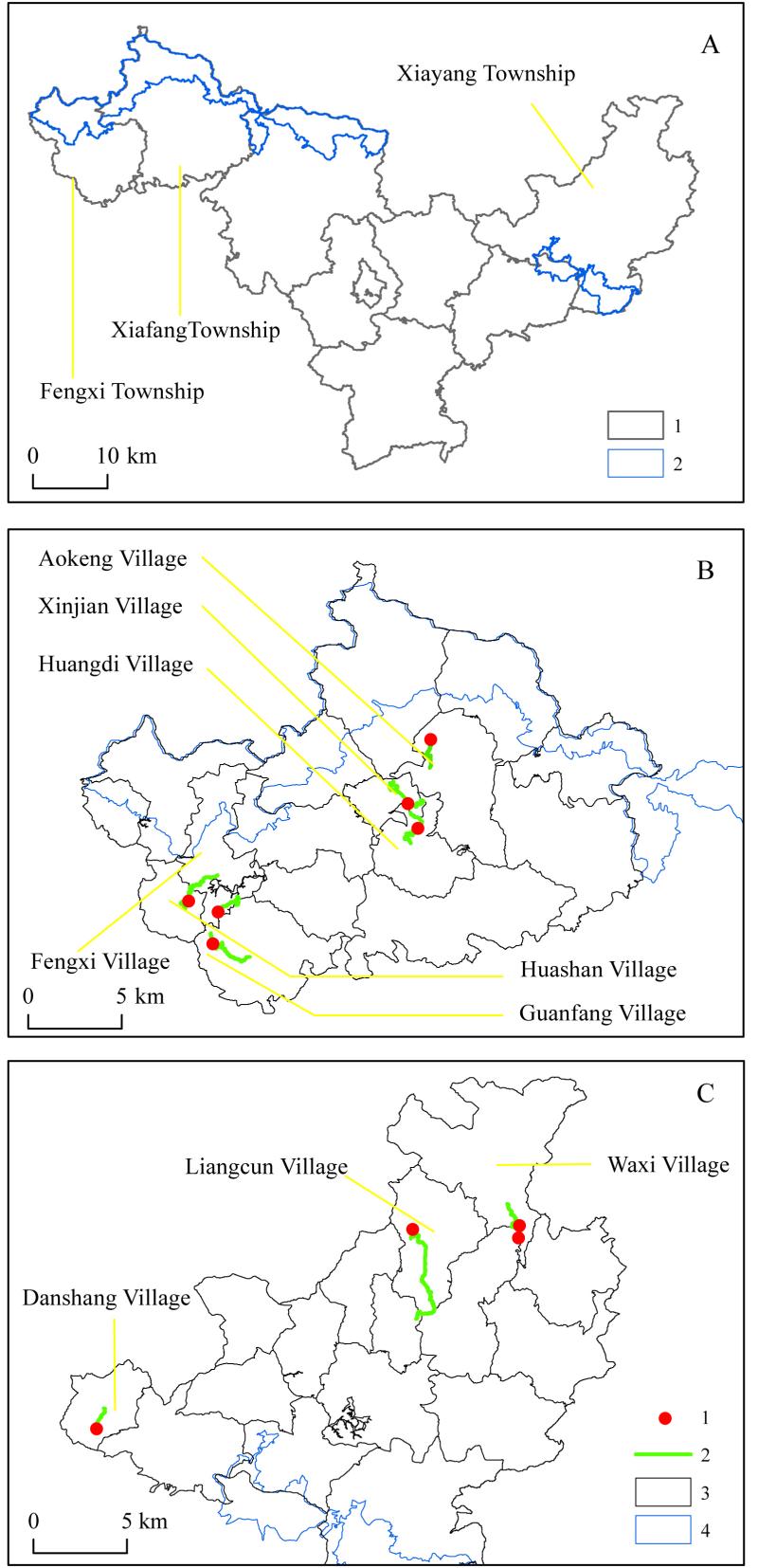


Figure S3. Field Survey Transects within Nine Administrative Villages. A) Depiction of townships and protected areas within Mingxi County, with '1' signifying township boundaries and '2' delineating the perimeters of protected areas; B) Configuration of six transects across six respective Administrative Villages; C) Configuration of three transects across three respective Administrative Villages. Notation '1' pinpoints all identified pangolin burrow sites ascertained through the transects. The legends for panels B and C are consistent: '1' indicates the pinpointed locations of detected burrows, '2' denotes the survey transects, '3' identifies the administrative village boundaries, and '4' demarcates the limits of protected areas.
