# Supplementary material for: Field survey data for conservation: Evaluating suitable habitat of Chinese pangolin at the county‐level in eastern China (2000–2040)
Source: Ecol Evol. 2024 Jun 3;14(6):e11512. doi: 10.1002/ece3.11512 (PMC11147814; doi:10.1002/ece3.11512)
Supplement: Supplementary file 2 — Appendix S2. [file ECE3-14-e11512-s001.docx]

Methods

Line transect method is one of the classic methods in wildlife macroecology research, widely used for field surveys of various mammals. Among the 90 transects we surveyed, each was 1-2 km long and 5-10 m wide. We recorded the number of caves found within the transects, considering caves within 300 m distance as one cave during the survey (Peng, 2020).

D (The density of pangolins within each transect) = N (The number of pangolins on that transect) /(L (transect length)×W(transect width)). D1 (average density of pangolins within the survey area) = ( ∑ D)/n (total number of transects).We estimated the population size of Chinese pangolins in highly suitable habitats. N (population size)=D1×S (suitable habitat area).

Results

In the 90 transects, a total of 106 Chinese pangolin caves were discovered, with an average density of 0.21 individuals per square kilometer and an estimated population size of approximately 10 individuals.

References

Peng, J. 2020. Study on the ecological geographical distribution，habitat selection and wild resources of Manis pentadactyla, Chongqing Normal University, China.
