# Supplementary material for: Field survey data for conservation: Evaluating suitable habitat of Chinese pangolin at the county‐level in eastern China (2000–2040)
Source: Ecol Evol. 2024 Jun 3;14(6):e11512. doi: 10.1002/ece3.11512 (PMC11147814; doi:10.1002/ece3.11512)

Video: The video footage of the Chinese pangolins' activity at the burrow entrance.

Photographs: The Photographs of the Chinese pangolins' continuous occupancy of their burrows.


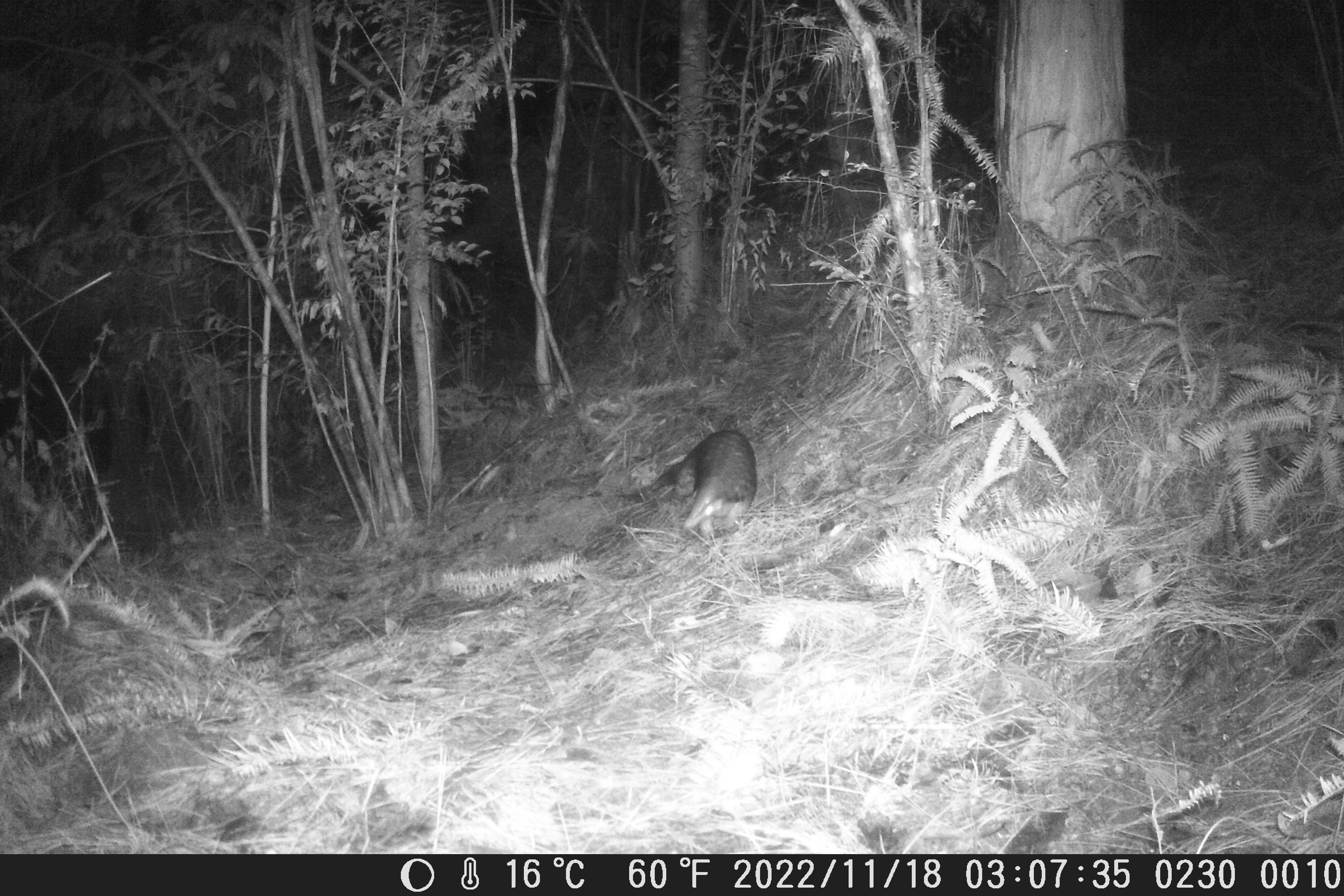


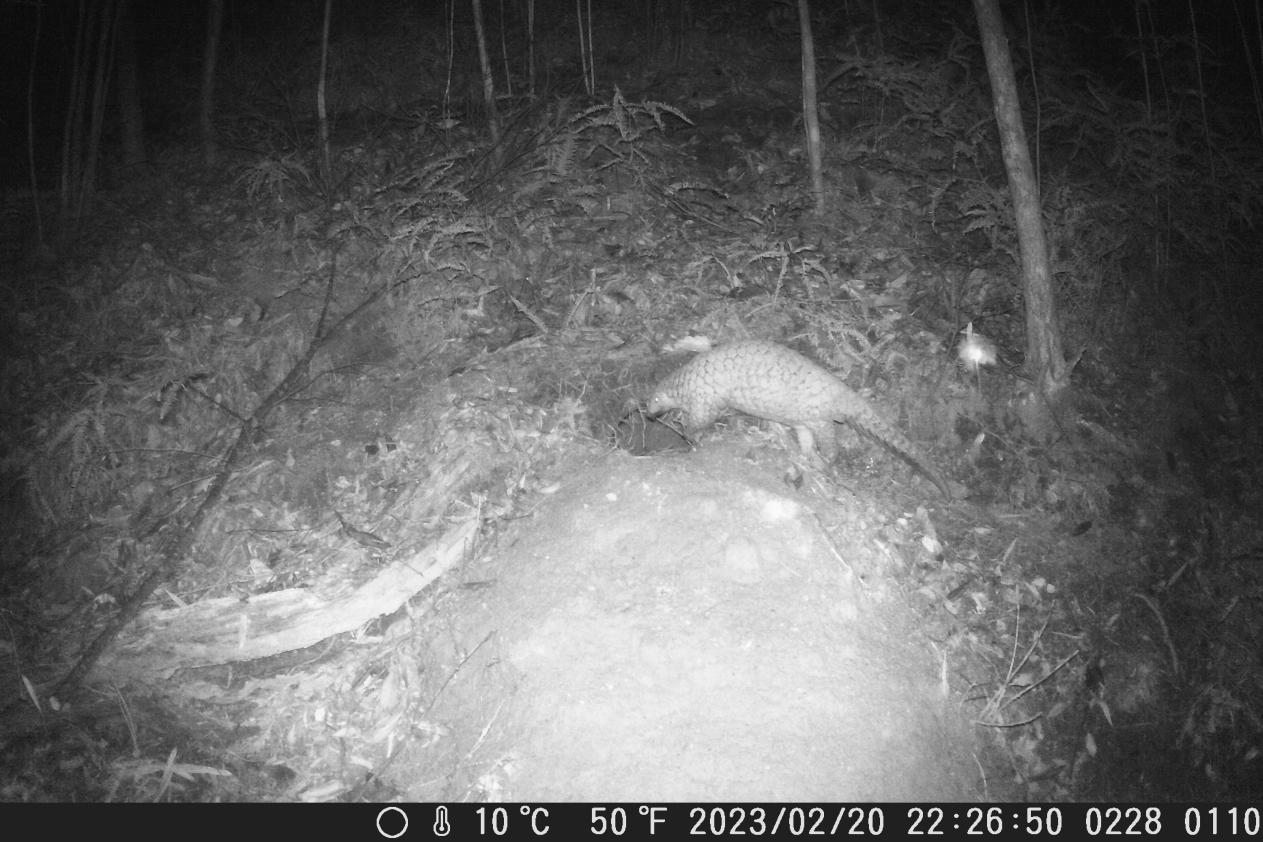

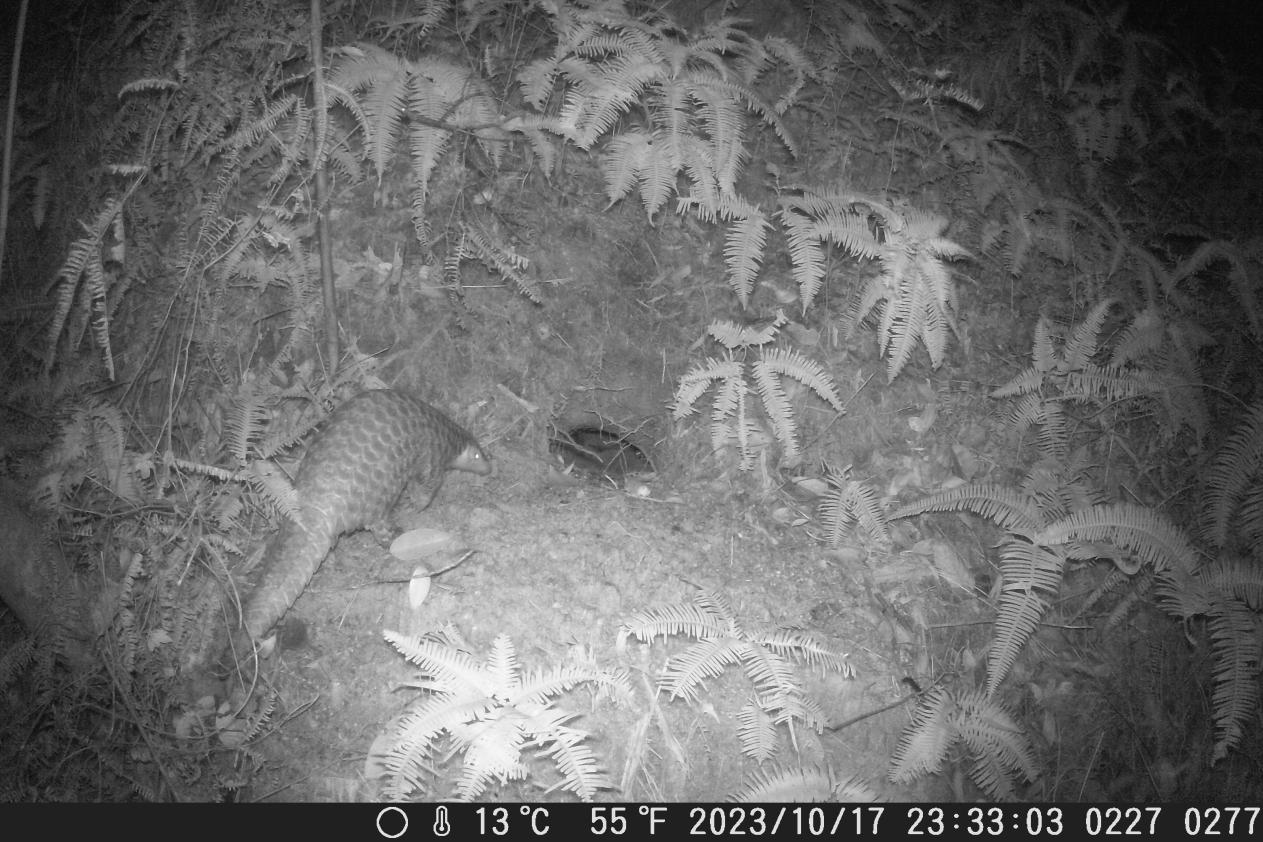

Supplement: Supplementary file 3 — Appendix S3. [file ECE3-14-e11512-s003.docx]
